# The Story of the Insane from Year to Year

**Published:** 1895-06-08

**Authors:** 


### THE STORY OF THE INSANE FROM YEAR TO YEAR.

Eighth Series.

### BROOKWOOD ASYLUM, SURREY.

Here there was an increase from 1,019 to 1,074, and the average number resident during 1894 was 1,054. It may be stated with the utmost confidence that this is the very highest number which any asylum should contain, and it is much to be wised that the Secretary of State for the Home Department would refuse his sanction to the erection of any asylum for a greater number.

Even at Brookwood there were during the year 278 admissions, and it must be no easy task for Dr. Barton to keep all these in his memory. There were 86 recoveries, 22 discharged relieved, and 104 deaths. The recoveries stand at 30·93 per cent. on the admissions, and the deaths at 9·86 on the average number resident. Of the deaths 59 were from brain diseases, 28 from thoracic affections, 2 from suicide, and 1 from arsenical poisoning. Not fewer than 78 of the year's admission owed their isanity to hereditary influence, and 38 to intemperance in drink. One of the patients who committed suicide was not suspected of suicidal tendency, and the jury very properly appended to their verdict a rider that no blame was attached to anyone. In the other instance the act was committed whe n the patient was absent on trial The case of poisoning was that of a man who had worked for ten years in the painter's shop—no doubt for his own benefit as well as for that of the asylum—had never shown any suicidal tendency, and then swallowed some green paint. Dr. Barton deplores the numerous changes in his staff of attendants and nurses, and he attributes these to the nature of the duties, and more especially to the strain entailed by the watching of suicidal cases. Dr. Barton adds that every inducement is offered to the efficient to remain in the asylum. It is, however, quite certain that higher wages and more leave of absence are not by any means invariably successful. We are inclined to think that the certainty of a pension at the age of fifty, with a bonus every fifth year, would do much in the right direction. The visitors say they "are well satisfied with the efficiency of the staff, and with the state and condition of the asylum."

### GARTNAVEL ASYLUM, GLASGOW.

During the year 1894 the number of patients was almost stationary, being 490 on January 1st and 495 on December 31st. The average number resident was 493, and the highest number reached was 505. There were 149 admissions, 47 recoveries, 66 discharged relieved, and 31 deaths. The percentage of recoveries on admissions was 31·5, and that of the deaths on the average number resident was 4·8. Fifteen of the deaths were from cerebral or spinal diseases, and 7 from consumption. Of the admissions, 22 were owing to alcoholic intemperance, and hereditary predisposition accounts for 8 only, which strikes us as being very low, and can hardly be accepted as reliable when we find 21 stated to be caused by previous attacks and 16 not ascertained. A considerable variety of professions was represented among the admissions. We notice artists, authors, army officers, chemists, engineers, medical men, manufacturers, teachers, &c., &c. Dr. Yellowlees mentions a somewhat rare occurrence in which recovery took place in a man who had been fed by means of the stomach tube three times daily for four years. There are two assistant medical officers in this asylum and two clinical clerks. One of the latter is a lady. As we believe this was the first journal in England to advocate the appointing of lady physicians in asylums, we are pleased to hear that Dr. Yellowlees can "testify strongly to the value of such an officer in the female division of an asylum."

### KILKENNY DISTRICT ASYLUM.

The limits of accommodation are given at 295 patients; but on January 1st 342 were in residence, and at the end of the year the number had risen to 346. There were 56 admissions, 17 recoveries, 5 discharged relieved, and 26 deaths. The recoveries stand at 30·3 per cent. on the admissions, and the deaths at 7 5 on the average number resident. Hereditary influence caused the insanity in 14 of the admissions, and intemperance in drink in 5. As pneumonia was the cause of death in 13 of the 26 deaths, it would seem as if this disease existed in epidemic form. There was also a slight outbreak of typhoid fever, and one death was indirectly due to it. Extensive alterations are going on in various parts of the asylum, and as Dr. Myles remarks, these are productive of much inconvenience, and interfere with the successful treatment of the patients; and the Commissioners say that "every praise is due to the staff for the patient manner in which they bear the great discomfort resulting from the disturbed state of the establishment, as both nurses and attendants have to sleep amongst the patients in the dormitories, even in those occupied by the most troublesome and objectionable lunatics." In English asylums this proceeding would probably cause a mutiny among the staff, but they take these things more easily in Ireland. The average cost per head per annum is £18 17s.

